# Role of interleukins in the detection of neonatal sepsis: a network meta-analysis

**DOI:** 10.3389/fped.2023.1267777

**Published:** 2023-11-02

**Authors:** Wei Xing, Ying Wang, Jiao Liu, Jie Pei, Chengyong Yu

**Affiliations:** ^1^ Institute for Central Laboratory, Weihai Central Hospital, Weihai, China; ^2^ Department of Laboratory Medicine, Weihai Central Hospital, Weihai, China

**Keywords:** interleukins, neonates, sepsis, detection, network meta-analysis

## Abstract

**Objectives:**

The purpose of the network meta-analysis was to make a more comprehensive comparison of different interleukins in the detection of neonatal sepsis and to pose clues in the field of clinical practice.

**Methods:**

Electronic databases of PubMed, Web of Science and Embase were systematically searched. Eligible studies included diagnostic tests utilizing interleukins to detect neonatal sepsis. We calculated pooled sensitivity, specificity, positive Likelihood Ratio (PLR) and negative Likelihood Ratio (NLR), diagnostic odds ratio (DOR), and superiority index.

**Results:**

Fifteen studies including 1,369 neonates diagnosed of sepsis were included in this meta-analysis. For the detection of early-onset sepsis in neonates, the pooled sensitivity was 0.91 (95% CI: 0.81, 0.97; *I*^2 ^= 0%, *p* = 0.946) and the pooled specificity was 0.98 (95% CI: 0.87, 0.97; *I*^2 ^= 46.3%, *p* = 0.172) for IL-8. For the detection of late-onset sepsis in neonates. the sensitivity was 0.96 (95% CI: 0.85, 1.00; *I*^2 ^= NA, *p* = NA) and the pooled specificity was 1.00 (95% CI: 0.92, 1.00; *I*^2 ^= NA, *p* = NA) for IL-27. Results of ANOVA model revealed that the superiority index of IL-6, IL-8, IL-10, and IL-27 were 1.20 (0.14, 5.00), 5.14 (0.33, 7.00), 0.75 (0.14, 5.00), and 1.31 (0.14, 5.00) in the detection of early-onset neonatal sepsis. Superiority index of IL-8, IL-10, and IL-27 were 1.84 (0.20, 5.00), 1.04 (0.20, 5.00), and 2.21 (0.20, 5.00) in the detection of late-onset neonatal sepsis.

**Conclusions:**

Findings of this network meta-analysis suggest that interleukins including IL-6, IL-8, IL-10, and IL-27 may have favorable performance in the detection of neonatal sepsis. IL-8 was more accurate in the detection of early-onset sepsis in neonates. IL-27 was more accurate in the detection of late-onset neonatal sepsis.

## Introduction

Sepsis is a life-threatening organ dysfunction associated with a dysregulated body's response to infection, it remains one of the most common causes of deaths in critically ill individuals (1, 2). Sepsis has been recognized as a global health priority in The World Health Assembly and WHO (3, 4) and thereafter became a public issue. The incidence of severe sepsis reached more than 300/100,000 in the United States (5). In addition, it is estimated that approximately 2.8 million deaths are attributable to sepsis in high-resource countries every year. Sepsis is related to most deaths due to chest infections in neonates and infants in Africa and Asia, the morbidity of neonatal sepsis in premature infants and very low birth weight infants is significantly higher, while the mortality rate is inversely proportional to the gestational age, the mortality rate of premature infants or young infants is higher than that of full-term infants (6, 7). Subsequently, early and accurate diagnosis of sepsis plays an important role in successful treatment and improving survival rate of septic patients. However, the signs and symptoms of neonatal sepsis are similar to those with non-infectious inflammation, which brings more difficulties to clinical diagnosis when the source of infection cannot be determined (8, 9).

Blood culture is still the irreplaceable gold standard for sepsis diagnosis, which can identify pathogens and perform antibiotic sensitivity tests to guide the treatment of bacterial infections, although it is a time-consuming program, with a high false negative rate, especially after the use of antibiotics (10). In addition, several biomarkers were used to achieve the early detection of sepsis. Conventional test approaches, including C-reactive protein and procalcitonin, have been proved less inaccurate in detecting sepsis in previous meta-analyses (11, 12). Interestingly, host immune responses including cytokines and chemokines during neonatal sepsis may help to detect and evaluate the severity of sepsis (13). Activation of pathogen recognition receptor (PRR) leads to the production of inflammatory mediators, such as interleukin-11β (IL-1β), IL-6, IL-8, IL-12, IL-18, interferon-γ (INF-γ) and tumor necrosis factor-α (TNF-α) (14). Expression of transforming growth factors-β (TGF-β), IL-4, IL-10, IL-11, IL-13 and other anti-inflammatory cytokines are programed to manipulate and balance inflammation (15). The diagnostic validity of IL-6, IL-8, IL-10 and IL-27 for neonatal sepsis were investigated in several cross-sectional studies and meta-analyses (16–24). Nevertheless, the sample sizes of these studies were relatively limited and results are heterogeneous. We performed this network meta-analysis to compare the performance of different interleukins in the detection of sepsis and to provide potential evidence for future research and clinical practice.

## Methods

The meta-analysis was conducted under the guidance of the Preferred Reporting Items for Systematic Reviews and Meta-analysis (PRISMA) (25). This review was not registered in the Cochrane database. All included studies had declared ethical approvals in the original articles, no ethical approval was necessary for this study. Patients or the public were not involved in any aspect of this study.

### Literature search and study selection

We performed a comprehensive search of PubMed, Web of Science, and Embase from inception to 31 January 2023. Only English language was considered. The following key terms were used for the database research: “Interleukin”, “IL”, “neonatal”, “neonate”, “sepsis”, “pyemias”, “pyohemias”, “septicemia”, “septic shock”, “septicemias”, and “poisoning, blood”. The references of related reviews were also screened for any possibly eligible tests. Inclusion criteria were as follows: (1) Different interleukins were used to detect early-onset or late-onset neonatal sepsis; (2) Absolute number of neonates with true positive (TP), false positive (FP), false negative (FN), and true negative (TN) were reported to form a 2 × 2 table or these outcomes could be calculated based on other reported indices; (3) Blood culture was used for the confirmation of neonatal sepsis. Exclusion criteria included: (1) Data to be analyzed cannot be extracted or calculated; (2) Case reports, review, letters, news, conference abstracts, animal studies, and animal experiments; (3) There was duplication or overlap in the research participants. If studies were done by the identical research group, those with the largest sample size or the most detailed data were included. Two independent researchers undertook the literature search and study screening. Disagreements were resolved through discussion.

### Data extraction and quality assessments

Two investigators independently performed the title and abstract screening of citations on the basis of the inclusion criteria mentioned above. Then a full-text evaluation of the studies was conducted for the final inclusion. Moreover, the following information of each study included was extracted or calculated: first author's name, year of publication, country of participants, number of participants, cut-off level, type of interleukin, absolute numbers of participants evaluated as TP, FP, TN, FN under interleukin test. The updated quality assessment of diagnostic accuracy studies (QUADAS-2) tool was used as an assessment of methodological quality, study validity, and risk of bias within the study (26). This tool includes a list of probable sources of bias for diagnostic accuracy studies in the context of patient selection, index test, reference standards, flow and timing (26).

## Statistical analysis

We used Meta-Disc software (Version 1.4) and the R (Version 4.1.2) for statistical analyses. A *p* value < 0.05 was considered to be statistically significant. Pooled sensitivity, specificity, positive Likelihood Ratio (PLR) and negative Likelihood Ratio (NLR), diagnostic odds ratio (DOR) and their 95% confidence intervals (CIs) were calculated using the random effects models. The Cochran *Q* test and the *I*^2^ statistics were introduced to qualitatively and quantitatively assess the heterogeneity between included studies. Insignificant, low, moderate, and high heterogeneity were identified if *I*^2^ values were 0%–25%, 25%–50%, 50%–75%, and 75%–100%, respectively (27). Furthermore, a Bayesian network meta-analysis (NMA) was performed to compare the diagnostic performance of different interleukins. Posterior estimates of absolute sensitivity, absolute specificity, diagnostic odds ratio, relative sensitivity, relative specificity and their corresponding 95% credible intervals were calculated using the two-way analysis of variance (ANOVA) model (28). Each analysis was based on noninformative priors for effect sizes and precision. Convergence and lack of autocorrelation were confirmed after 2 chains and a 1,000 simulation burn-in phase. Afterwards, direct probability statements were derived from an additional 10,000 simulation phase (29). The superiority of a diagnostic test could be quantified using a superiority (S) index, which ranges from 0 to ∞ with S tending to ∞ and S tending to 0 as the tests is superior and inferior increases, respectively, and S tending to 1 the more the tests are equal (30). Funnel plots were proposed to assess potential bias of publication. The Deeks' method was used to statistically check the asymmetry of the funnel plot and detect publication bias. Besides, we conducted sensitivity analysis to evaluate the impacts of single study on the overall outcomes.

## Results

### Study selection and characteristics

**Figure 1:**
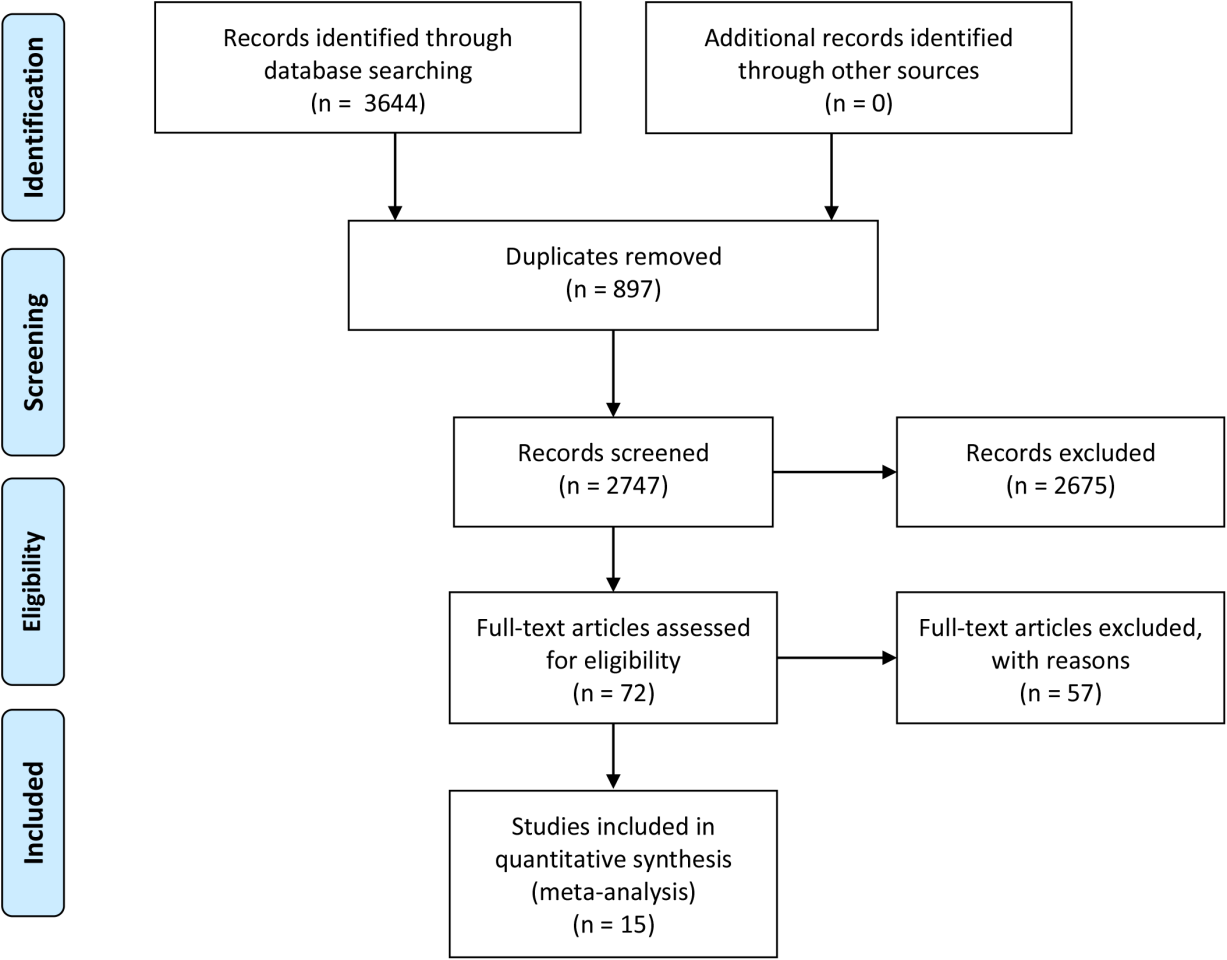
Flowchart of the literature search.

**Figure 2:**
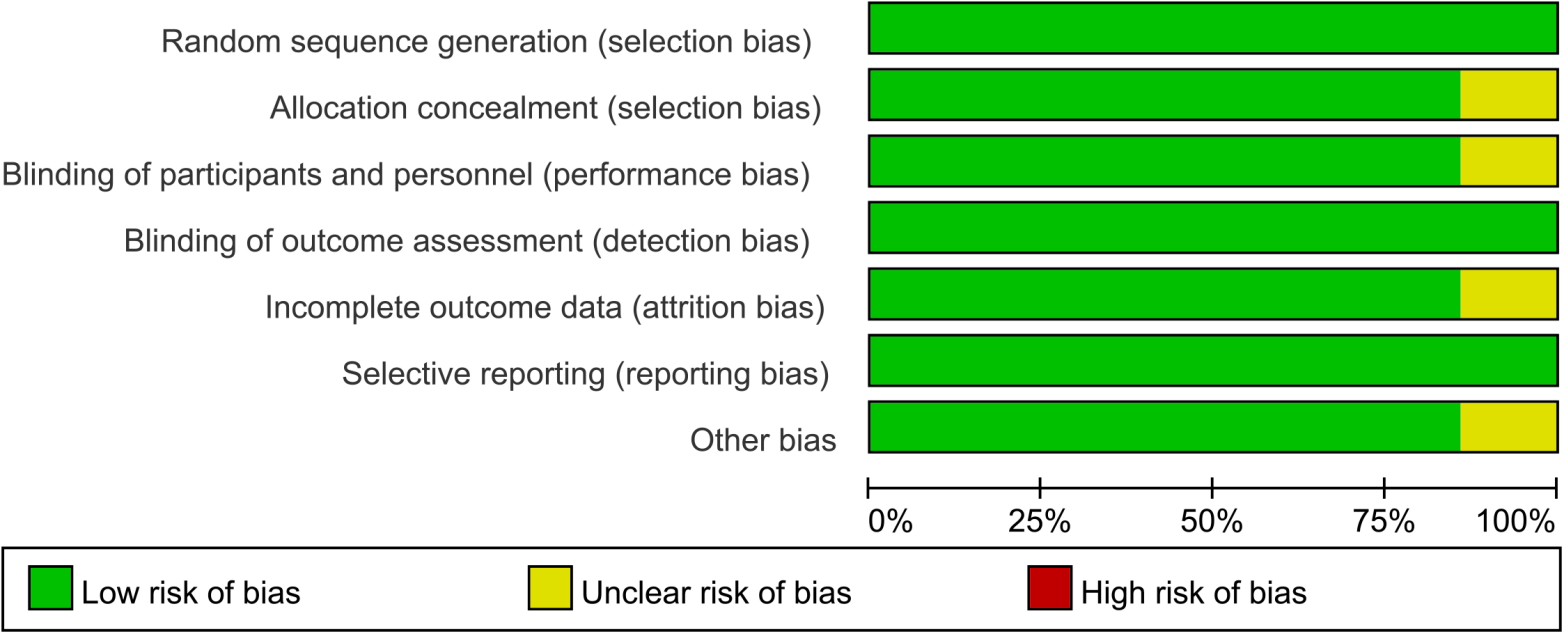
Summary of risk of bias in studies included.

A total of 3,644 articles were identified from the databases search, in which 897 duplicates were removed and 2,747 studies were excluded through an initial screening. Based on the full text assessment for eligibility of the remaining 72 citations, 15 studies with 1,369 neonates were identified for inclusion in this meta-analysis (22, 31–44). No additional studies were found through bibliography screening of the relevant reviews. Eleven enrolled studies were performed among patients with early-onset sepsis, four were in late-onset sepsis (Table 1). Years of publication of included studies ranged from 1998 to 2022. The included studies were done in 9 countries including China, Colombia, Denmark, Egypt, Finland, Germany, Iran, Spain, and Turkey (Table 1). Figure 1 shows the detailed flow of the literature search and study selection. Risk of bias for each included study were rated as low (Figure 2).

**Table 1:** Study characteristics.

Author	Year	Country	Number of participants	Sepsis diagnosis	Type of sepsis	Type of interleukin	cut-off (pg/ml)	TP	FP	FN	TN
Bender	2014	Denmark	123	Culture, laboratory evaluation	early onset	IL-10	15	19	20	10	74
Berner	1998	Germany	101	Culture, clinical, laboratory evaluation	early onset	IL-8	300	32	5	3	61
Boskabadi	2010	Iran	80	Culture, clinical evaluation	late onset	IL-8	60	36	0	2	42
Canpolat	2011	Turkey	74	Culture, clinical, laboratory evaluation	early onset	IL-6	7.6	30	1	2	41
Cetin	2014	Turkey	40	Culture, clinical, laboratory evaluation	early onset	IL-6	11	9	11	1	19
Cobo	2013	Spain	176	Culture, clinical, laboratory evaluation	early onset	IL-6	38	10	30	2	134
Cortés	2020	Colombia	93	Culture, clinical, laboratory evaluation	early onset	IL-6	2.38	40	24	7	22
Ebenebe	2019	Germany	67	Culture, laboratory evaluation	early onset	IL-6	40	20	11	7	29
Fahmy	2020	Egypt	84	Culture, clinical, laboratory evaluation	early onset	IL-27	NS	44	7	3	30
Gharehbaghi	2008	Iran	45	Culture, clinical, laboratory evaluation	early onset	IL-6	20	8	4	9	24
He	2017	China	151	Culture, clinical, laboratory evaluation	early onset	IL-27	1,000	48	24	20	59
Ng	2007	China	155	Culture, clinical, laboratory evaluation	late onset	IL-10	7.6	37	18	7	93
Nupponen	2001	Finland	35	Culture, clinical evaluation	early onset	IL-8	50	20	0	2	13
Omran	2021	Egypt	55	Culture, clinical evaluation	late onset	IL-10	33.6	26	6	1	22
Tosson	2022	Egypt	90	Culture, clinical evaluation	late onset	IL-27	283.8	44	0	2	44

TP, true positive; FP, false positive; FN, false negative; TN, true negative; NS, not specified.

### Detection performance for early-onset sepsis

For the detection of early-onset sepsis in neonates. Six studies reported detection outcomes of IL-6 in the detection of sepsis, the pooled sensitivity was 0.81 (95% CI: 0.73, 0.87; *I*^2 ^= 68.8%, *p* = 0.007) and the pooled specificity was 0.77 (95% CI: 0.72, 0.81; *I*^2 ^= 87.5%, *p* < 0.001), respectively. PLR, NLR, and DOR were 3.17 (95% CI: 1.85, 5.44), 0.27 (95% CI: 0.14, 0.54), and 13.90 (95% CI: 4.85, 39.85) (Table 2A). Two studies reported outcomes of IL-8 in the detection of sepsis, the pooled sensitivity was 0.91 (95% CI: 0.81, 0.97; *I*^2 ^= 0%, *p* = 0.946) and the pooled specificity was 0.98 (95% CI: 0.87, 0.97; *I*^2 ^= 46.3%, *p* = 0.172), respectively. PLR, NLR, and DOR were 12.87 (95% CI: 5.72, 28.94), 0.10 (95% CI: 0.05, 0.23), and 143.74 (95% CI: 37.38, 552.75) (Table 2A). One study reported outcomes of IL-10 in the detection of sepsis, the sensitivity was 0.66 (95% CI: 0.46, 0.82; *I*^2 ^= NA, *p* = NA) and the specificity was 0.79 (95% CI: 0.69, 0.87; *I*^2 ^= NA, *p* = NA), respectively. PLR, NLR, and DOR were 3.08 (95% CI: 1.93, 4.93), 0.44 (95% CI: 0.26, 0.73), and 7.03 (95% CI: 2.83, 17.49) (Table 2A). Two studies reported outcomes of IL-27 in the detection of sepsis, the pooled sensitivity was 0.80 (95% CI: 0.72, 0.87; *I*^2 ^= 94.0%, *p* = 0.001) and the pooled specificity was 0.74 (95% CI: 0.65, 0.82; *I*^2 ^= 28.0%, *p* = 0.239), respectively. PLR, NLR, and DOR were 3.29 (95% CI: 1.63, 6.67), 0.19 (95% CI: 0.04, 1.09), and 17.70 (95% CI: 1.74, 179.88) (Table 2A).

**Table 2A:** Performance of different interleukins in detecting early-onset neonatal sepsis.

Test	Number of studies	Sensitivity (95% CI)	Specificity (95% CI)	PLR (95% CI)	NLR (95% CI)	DOR (95% CI)
IL-6	6	0.81 (0.73, 0.87)	0.77 (0.72, 0.81)	3.17 (1.85, 5.44)	0.27 (0.14, 0.54)	13.90 (4.85, 39.85)
IL-8	2	0.91 (0.81, 0.97)	0.94 (0.87, 0.98)	12.87 (5.72, 28.94)	0.10 (0.05, 0.23)	143.74 (37.38, 552.75)
IL-10	1	0.66 (0.46, 0.82)	0.79 (0.69, 0.87)	3.08 (1.93, 4.93)	0.44 (0.26, 0.73)	7.03 (2.83, 17.49)
IL-27	2	0.80 (0.72, 0.87)	0.74 (0.65, 0.82)	3.29 (1.63, 6.67)	0.19 (0.04, 1.09)	17.70 (1.74, 179.88)

### Detection performance for late-onset sepsis

For the detection of late-onset sepsis in neonates. Two studies reported outcomes of IL-8 in the detection of sepsis, the pooled sensitivity was 1.00 (95% CI: 0.94, 1.00; *I*^2 ^= NA, *p* = NA) and the pooled specificity was 0.05 (95% CI: 0.01, 0.18; *I*^2 ^= NA%, *p* = NA), respectively. PLR, NLR, and DOR were 1.06 (95% CI: 0.97, 1.15), 0.13 (95% CI: 0.01, 2.59), and 8.29 (95% CI: 0.39, 177.47) (Table 2B). Two studies reported outcomes of IL-10 in the detection of sepsis, the pooled sensitivity was 0.89 (95% CI: 0.79, 0.95; *I*^2 ^= 65.3%, *p* = 0.090) and the pooled specificity was 0.83 (95% CI: 0.75, 0.89; *I*^2 ^= 0%, *p* = 0.523), respectively. PLR, NLR, and DOR were 4.98 (95% CI: 3.42, 7.26), 0.13 (95% CI: 0.04, 0.45), and 34.24 (95% CI: 13.23, 88.64) (Table 2B). One study reported outcomes of IL-27 in the detection of sepsis, the sensitivity was 0.96 (95% CI: 0.85, 1.00; *I*^2 ^= NA, *p* = NA) and the pooled specificity was 1.00 (95% CI: 0.92, 1.00; *I*^2 ^= NA, *p* = NA), respectively. PLR, NLR, and DOR were 85.21 (95% CI: 5.41, 1,342.6), 0.05 (95% CI: 0.02, 0.18), and 1,584.2 (95% CI: 73.93, 33,945.3) (Table 2B).

**Table 2B:** Performance of different interleukins in detecting late-onset neonatal sepsis.

Test	Number of studies	Sensitivity (95% CI)	Specificity (95% CI)	PLR (95% CI)	NLR (95% CI)	DOR (95% CI)
IL-8	1	1.00 (0.94, 1.00)	0.05 (0.01, 0.18)	1.06 (0.97, 1.15)	0.13 (0.01, 2.59)	8.29 (0.39, 177.47)
IL-10	2	0.89 (0.79, 0.95)	0.83 (0.75, 0.89)	4.98 (3.42, 7.26)	0.13 (0.04, 0.45)	34.24 (13.23, 88.64)
IL-27	1	0.96 (0.85, 1.00)	1.000 (0.920, 1.000)	85.21 (5.41, 1,342.6)	0.05 (0.02, 0.18)	1,584.2 (73.93, 33,945.3)

### ANOVA model for NMA

Results of ANOVA model revealed that the superiority index of IL-6, IL-8, IL-10, and IL-27 were 1.20 (0.14, 5.00), 5.14 (0.33, 7.00), 0.75 (0.14, 5.00), and 1.31 (0.14, 5.00) in the detection of early-onset neonatal sepsis. Superiority index of IL-8, IL-10, and IL-27 were 1.84 (0.20, 5.00), 1.04 (0.20, 5.00), and 2.21 (0.20, 5.00) in the detection of late-onset neonatal sepsis. Results on the posterior estimates and their respective 95% credible intervals are shown in Tables 3A,B.

**Table 3A:** Posterior estimates of detection performance of different interleukins in early-onset neonatal sepsis.

Test	Number of studies	Absolute sensitivity			Absolute specificity			Diagnostic odds ratio				Superiority index			
		Mean	Lower	Upper	Mean	Lower	Upper	Mean	Lower	Upper	Rank	Mean	Lower	Upper	Rank
IL-6	6	0.78	0.63	0.88	0.74	0.58	0.84	12.03	3.45	28.42	4	1.20	0.14	5.00	3
IL-8	2	0.85	0.54	0.98	0.90	0.63	0.99	250.95	5.60	1,392.90	1	5.14	0.33	7.00	1
IL-10	1	0.61	0.20	0.92	0.69	0.23	0.95	13.95	0.23	80.55	3	0.75	0.14	5.00	4
IL-27	2	0.78	0.48	0.94	0.69	0.36	0.91	17.81	1.10	81.32	2	1.31	0.14	5.00	2

**Table 3B:** Posterior estimates of detection performance of different interleukins in late-onset neonatal sepsis.

Test	Number of studies	Absolute sensitivity			Absolute specificity			Diagnostic odds ratio				Superiority index			
		Mean	Lower	Upper	Mean	Lower	Upper	Mean	Lower	Upper	Rank	Mean	Lower	Upper	Rank
IL-8	1	0.74	0.24	0.96	0.87	0.44	1.00	32,94,169.58	0.73	14,31,348.44	1	1.84	0.20	5.00	2
IL-10	2	0.80	0.47	0.92	0.77	0.44	0.93	45.27	1.73	227.99	3	1.04	0.20	5.00	3
IL-27	1	0.77	0.25	0.97	0.89	0.49	1.00	980,190.30	0.40	15,26,342.50	2	2.21	0.20	5.00	1

### Publication bias

Deeks' funnel plot asymmetry test was not performed because the number of included studies in each meta-analysis was <10.

### Sensitivity analysis

The sensitivity analysis was performed to evaluate the impacts of individual study on the overall results. No outlier was identified in all sensitivity analyses.

## Discussion

Neonatal sepsis remains one of the leading sources of morbidity and mortality in the neonatal intensive care unit (NICU) (45). Owing to the variable and non-specific signs and symptoms, the diagnosis and treatment of neonatal sepsis is still a challenging task (46). Active management rather than prospective management can be adopted to reduce the possibility of accidental use of antibiotics, treatment costs and over treatment (47).

Previous studies have demonstrated that cytokines such as IL-6, IL-8, IL-10, and IL-27 were biomarkers of neonatal sepsis and their diagnostic properties have been investigated. However, the outcomes of these investigations are heterogeneous. The primary aim of this study was to explore the diagnostic performance of different subtypes of interleukins by pooling the evidence in published articles.

The level of IL-6 in healthy people is very low, generally not more than 7 pg/ml, while the level of IL-6 in serum of septic patients increases rapidly in the early stage of infection, and can reach the peak within 2 h (48). Likewise, IL-8 regulates the migration and activation of leukocytes, and its level is rapidly assessed within 1–3 h after infection, with a half-life of less than 4 h (19). Furthermore, IL-10 is expressed by many innate and adaptive immune response cells, thus it plays an important role in the early diagnosis of sepsis in neonates (49). In recent years, IL-27 has been used as a biological marker for sepsis diagnosis (20, 22, 40, 44). Results of conventional meta-analysis showed that IL-8 manifested the highest pooled sensitivity and specificity in the detection of early-onset sepsis in neonates, which indicates the superior diagnostic properties of IL-8 for neonates as compared to IL-6, IL-8, IL-10. In the detection of late-onset neonatal sepsis, IL-8 showed the highest sensitivity, and IL-27 demonstrated the highest specificity. Moreover, these aforementioned results were consistent with those of the network meta-analysis using the ANOVA model. IL-8 ranked the best in the detection of early-onset sepsis which may be associated with its short half-life (19). The detection performance of IL-27, IL-8, and IL-10 in late-onset sepsis ranked from superior to inferior based on the superiority index. Studies have shown that the effect of IL-27 may be related to the pathogen that infects the bacteria, the host's immune status, and the duration of infection, a better understanding of pathogen specific IL-27 responses during sepsis may have clinical benefits (50). The underlying mechanism needs to be furtherly investigated. However, results of this study may provide evidence for clinical application with regard to the choice of different subtypes of interleukins for the diagnosis of neonatal sepsis. Moreover, it was proved in He's et al. study that the combined use of IL-27 and PCT (AUC = 0.792) revealed greater performance than PCT or IL-27 alone in the detection of neonatal sepsis (40). Zeitoun's et al. study showed that the combination was IL-10 and nCD64 together provided sensitivity of 95% and specificity of 83% in the detection of neonatal sepsis (51). The potential roles of interleukins in combination with other inflammatory markers are promising and need to be further investigated.

This is the first network meta-analysis of detection test accuracy on neonatal sepsis. Compared to conventional meta-analysis, network meta-analysis holds the process of combining and summarizing direct and indirect evidence from independent studies to assess the diagnostic accuracy of different tests for the same disease. Network meta-analysis provides a unified reasoning framework and uses data more effectively. Superiority index was introduced to quantify the superiority of a specific interleukin in the diagnosis of neonatal sepsis. In this study, conventional meta-analysis was also performed, we firstly endeavored to complete a detailed and comprehensive literature research of electronic databases as to retrieve as much related studies as we can. Two independent reviewers screened the titles, abstracts and full-text of the articles and undertook the process of data extraction. In addition, heterogeneity in studies include were assessed. The detection properties of interleukins in the detection of early-onset sepsis and late-onset sepsis were performed. Sensitivity analysis indicated that the pooled outcomes were robust after omitting study one after another in this meta-analysis. We planned to perform subgroup analysis and meta-regression to explore the potential source of heterogeneity in studies enrolled, nevertheless, we were not able to complete these analyses due to the limited number of studies eligible for the inclusion criteria. A meta-analysis based on individual patient data is warranted to further address the issue on the optimal cut-offs of different interleukins in the detection of early-onset or late-onset sepsis. Moreover, although considered the gold standard for detecting sepsis, it is reported that the positive rate of blood culture in infant cases is as low as 3.3% (52), which may cause potential bias in this study.

## Conclusion

Interleukins including IL-6, IL-8, IL-10, and IL-27 demonstrated favorable performance in the detection of neonatal sepsis. IL-8 showed the most optimal properties in the diagnosis of early-onset sepsis in neonates as compared to IL-6, IL-8, IL-10. IL-27 revealed the most superior validity in the detection of late-onset sepsis. Evidence on the detection performance of combined biomarkers including interleukins is warranted to pose indications for clinical practitioners.

## Data Availability

The original contributions presented in the study are included in the article/Supplementary Material, further inquiries can be directed to the corresponding author.
